# Acute and Subchronic Toxicological Evaluation of the Herbal Product HAD-B1 in Rats

**DOI:** 10.1155/2021/9970822

**Published:** 2021-05-31

**Authors:** So-Jung Park, Soo-Dam Kim, Eun-Bin Kwag, Ji Hye Park, Hwa-Seung Yoo

**Affiliations:** ^1^East West Cancer Center, Daejeon Korean Medicine Hospital, Daejeon University, 75, 176 Bun-Gil, Daedeok-daero, Seo-gu, Daejeon, 35-235, Republic of Korea; ^2^East West Cancer Center, Seoul Korean Medicine Hospital, Daejeon University, 32, Beobwon-ro 11-gil, Songpa-gu, Seoul 05-836, Republic of Korea

## Abstract

This study evaluates acute and subchronic toxicity of a Korean herbal formula HAD-B1 in rat to investigate whether HAD-B1 has potential toxicity to humans. First, the study to assess the acute oral toxicity at dose levels of 0, 500, 1000, and 2000 mg/kg body weight (BW) was performed in male and female SD rats (Crl: CD, specific pathogen-free) (*n* = 5/group). Based on the result of the acute oral study, 4 weeks' dose range finding study and 13 weeks' subchronic study were performed (dose range finding study, DRF; *n* = 5/group) and 13 weeks (subchronic study; *n* = 10/group) in male and female SD rats. The control group was administered with distilled water (DW). Clinical signs, body weight, food consumption, ophthalmic examination, urinalysis, hematological/biochemical parameters, gross finding at necropsy, and histopathological examination were investigated and recorded. In the oral acute toxicity study of SD rats, no clinical signs, mortality, body weight changes, and gross findings were observed. Also, there were no treatment-related changes in the 4-week DRF study. Based on these results, a 13-week repeated-dose toxicity study (subchronic) in SD rats was performed. HAD-B1 showed temporal hypersalivation in clinical signs and an increased tendency in body weight at 2000 mg/kg BW. However, there were no treatment-related changes in mortality, food consumption, ophthalmology, urinalysis, hematology, biochemistry, gross finding at necropsy, organ weights, and histopathology in either sex of any group. Based on this toxicological evaluation of HAD-B1, we concluded that no target organ was determined, and the no observed adverse effect level (NOAEL) of HAD-B1 was determined to be > 2000 mg/kg B W. Therefore, we decided that consuming HAD-B1 is relatively nontoxic.

## 1. Introduction

Lung cancer is defined as the uncontrolled growth of cells originating in the lungs [1]. There are different types of lung cancer, non-small-cell lung cancer (NSCLC) and small-cell lung cancer (SCLC). NSCLC accounts for 80–85% of all lung cancers, while SCLC is much infrequent, consisting of 10–15% of all lung cancers [2]. Lung cancer is the most common malignancies world. In Korea, lung cancer is the number one cause of cancer-related mortality (NCCN guidelines, 2012). Therefore, efforts to decrease the lung cancer mortality rate are an important problem to solve. Advances in molecular biology have led to the discovery of various genetic mutations associated with the pathogenesis of non-small-cell lung cancer. Various targeted therapies have been developed based on those findings, leading to breakthroughs in lung cancer treatments [3]. However, these targeted therapies seem to only work for early-stage lung cancer and have limitations such as drug resistance and high cost [4, 5]. Therefore, we must find alternative therapies that can help lung cancer patients with various stages of cancer.

CAM (complementary and alternative medicine) refers to therapies such as acupuncture, herbal medicine, tai chi, and they are considered complementary and alternative methods to conventional medical treatment. Even though CAM medicine was used longer than conventional medicine, it is still considered to lack scientific validity. Therefore, researchers worldwide are trying to build scientific data on the usage of CAM in helping cancer patients. The Daejeon University of Korean Medicine is one of them, and we have developed an herbal formula HAD-B1 to help lung cancer patients. Through previous research, HAD-B1 proved significant benefits in suppressing cancer cells' growth, reducing chemotherapy's side effects, and improving the quality of life of cancer patients by the formula itself or combined with the standard treatments [6, 7]. This present study aims to further investigate the efficacy of HAD-B1 by evaluating acute and subchronic (4 weeks, 13 weeks) toxicological evaluation of HAD-B1 in the rat.

## 2. Materials and Methods

### 2.1. Overview

The research protocols for this study were reviewed and assessed (IAC2015-0120, IAC2015-0418, IAC2015-1231) according to the regulation for the Institutional Animal Care and Use Committee at KTR based on the Laboratory Animal Act (Enforcement Date of July 30, 2013) (No. 11987 (July 30, 2013, partial revision)) and the Animal Protection Act (Enforcement Date of January 20, 2015) (No. 13023 (January 20, 2015, partial revision)). Also, all the tests performed in this paper are following Guidelines for toxicity testing of pharmaceuticals, Ministry of Food and Drug Safety, MFDS.

### 2.2. HAD-B1

**Figure 1:**
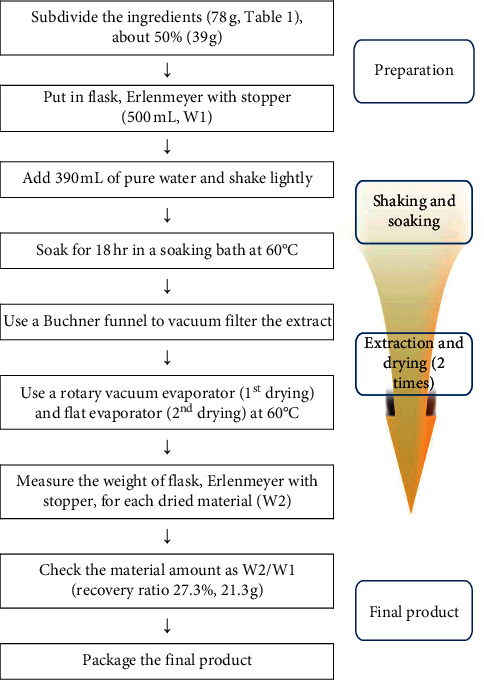
Process of HAD-B1 extraction.

HAD-B1 used for both acute and subchronic trials was provided by Kyoung Bang pharmaceutical Co. (Namdong-daero, Namdong-gu, Incheon Metropolitan City, Korea). The ingredients of HAD-B1 are listed in Table 1. In order to prepare HAD-B1 powder, 4 herbs were soaked for 18 hr at 60°C in a soaking bath. After the fluid extracts had been dried twice by using a rotary vacuum evaporator and a flat evaporator at 60°C, the powder (recovery ratio: 27.3%) was gained for the experiments (Figure 1). This dark brown powder (test substance, HAD-B1) was dissolved in distilled water (DW, Daihan Pharm Co., Ltd.) and suspended for oral administration.

### 2.3. 3D HPLC Analysis of HAD-B1

**Figure 2:**
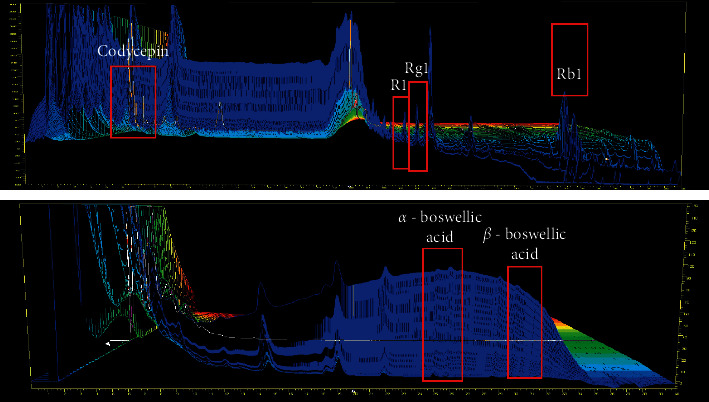
3D HPLC analysis results of HAD-B1. There are six compounds tested (cordycepin, R1, Rg1, Rb1, *α*-boswellic acid, *ß*-boswellic acid).

HAD-B1 was prepared by extracting HAD-B1 powder with 9.72 g of powder in 10 ml of methanol at room temperature. Then, it was centrifuged at 1000 × g for 30 min and filtered, applied to the C18 column, and eluted using acetonitrile mixed with DW. Then, we closely measured 10 mg of the standard brand of cordycepin, 10 mg of notoginsenoside R1, 10 mg of each ginsenoside Rg1, and Rb1 then diluted them with ethanol until the mixture reached exactly 100 ml, using this as the base liquid. Next, we measured 20 *μ*L of the test solution and base liquid each. We used these conditions to test using the liquid chromatography method, measuring each liquid's cordycepin, notoginsenoside R1, ginsenoside Rg1, and ginsenoside Rb1's peak area and finding the 3D HLPC. Closely measured 10 mg of alpha-boswellic acid and beta-boswellic acid was diluted with ethanol until the mixture measures exactly 100 ml and was used as the base liquid. Measured 20 *μ*L of the test solution and base liquid was used to test the liquid chromatography method, measuring each liquid's alpha-boswellic acid and beta-boswellic acid's peak area and finding the 3D HLPC. Six components were detected (Figure 2).

### 2.4. Experimental Animals and Housing Conditions

All of the rats used in tests presented in this experiment are SD rats (Crl: CD, specific pathogen-free) and Orient Bio Co., Ltd (Buk-myeon, Gapyeong-gun, Gyeonggi-do, Korea) supplied the animals. Room temperature was maintained at 22 ± 3°C, relative humidity of 50 ± 20%, 10 to 20 time air ventilation in an hour, and lux luminous intensity of 150 to 300 with a 12 hr light/dark cycle. Temperature and relative humidity were automatically monitored every 30 minutes by an automatic instrument, and environmental conditions were measured periodically during the examination period. The rats were fed with a pellet diet (Rodent diet 20 5053 (Labdiet, USA)), and given the R/O (reverse osmosis) water ad libitum.

### 2.5. Acute Oral Toxicity Test

#### 2.5.1. Grouping

According to the guideline (guidelines for toxicity testing of pharmaceuticals, Ministry of Food and Drug Safety, MFDS), the study was conducted. Forty-four 5-week-old rats (22 females, 22 males) were delivered. The rats had acclimation day of a week. After that, 40 6-week-old healthy rats were chosen. They were divided into 4 groups, including a control group (5 rats/sex/group) (Table 2).

#### 2.5.2. Dosage and Administration

Administration dosage was determined following the drug toxicity test standards of the Ministry of Food and Drug Safety. Therefore, the highest dose given was 2000 mg/kg, and median and the low dose were determined using a standard ratio of 2 (1000 and 500 mg/kg), respectively, with a volume of 10 mL/kg BW. The control group was administered with distilled water (Table 2). The rats had fasted for approximately 18 hr before the first dosing. The drug was delivered directly to the stomach using a sonde feeding needle catheter. Each animal was observed for signs of toxicity manifestations and behavioral changes at 0.5, 1, 2, 3, and 4 hr after the administration.

#### 2.5.3. Observations and Measurement

We inspected every rat once a day and 14 days after the administration. Weight measurement took place when at the initial import, before the grouping, before administrating, 7 and 14 days after the administration, and recorded every time. Fourteen days after the drug administration, a visual inspection was conducted and euthanized. Organs were checked with the naked eyes.

### 2.6. DRF (4-Week) and Subchronic Toxicity (13-Week) Study

The study was conducted according to the guideline (Guidelines for toxicity testing of pharmaceuticals, Ministry of Food and Drug Safety, MFDS).

#### 2.6.1. Grouping

In the DRF study, Orient Bio Co., Ltd (Buk-myeon, Gapyeong-gun, Gyeonggi-do, Korea) supplied 44 5-week-old rats (22 females, 22 males). The rats had one week of acclimation. After that, 40 6-week-old healthy rats were chosen for each study. We divided them into 4 groups, including a control group (5 rats/sex/group). The rats were divided into four groups: three treatment groups with different doses and 1 control group. In the subchronic study, 110 5-week-old rats (55 females and 55 males) were supplied from Orient Bio Co., Ltd (Buk-myeon, Gapyeong-gun, Gyeonggi-do, Korea). After a week of the acclimation period, 100 rats (50 females and 50 males) were selected and separated into 4 groups: 3 treatment groups and 1 control group.

#### 2.6.2. Dosage and Administration

The animals consumed DW and the substance of 0, 500, 1000, and 2000 mg/kg BW was tested, orally once a day for 4-week and 13-week (DRF study; 5 rats/sex/group, subchronic study; 10 rats/sex/group), respectively. Dosage administration for the subchronic test was determined to be the same based on the DRF study results, which showed toxic abnormalities. Therefore, it was determined that the dosage could be the same. Additionally, 10 rats (5 females, 5 males) were added to the control group and the highest dose group in order to evaluate the reversibility of the drug. In the subchronic study, the doses were chosen based on the results of the DRF study, and additional recovery animals (5 rats/sex) in the control and the high-dose group were included for observation of reversibility or persistence of any toxic effects after the treatment period. During the study period, all animals were observed once a day for clinical signs and mortality.

#### 2.6.3. Observation and Measurement

*(1) Body Weight and Food Consumption.* Weight measurement took place when at the initial import, before the grouping, before administrating, 7 and 14 days after the administration, and recorded every time. Furthermore, to find out relative organ weight, fasting weight was measured. The amount of food consumed was measured before drug administration and once every week after the administration has started. Differences in food given and food consumed were recorded per cage.

*(2) Ophthalmic Examination and Urinalysis.* The ophthalmological examination (Genesis, Kowa, Japan) and urinalysis were not performed for the DRF study and were performed during the last week of the treatment and recovery period for the subchronic study. Fresh urine was collected through the metabolic cage and used for urinalysis (specific gravity, pH, protein, glucose, ketone body, bilirubin, urobilinogen, nitrite, blood, and leukocyte), urine sedimentation, and color test. Urinalysis was performed using an automatic tester (CliniTek 500, Siemens, Germany), urine stick (Multistix 10 SG, Siemens, Germany), and a microscope (Leica, Germany) for urine sediment test. An ophthalmic test was performed for 10 rats (5 females, 5 males) per group with naked eyes.

*(3) Gross Finding at Necropsy.* All animals were fasted overnight and anesthetized by isoflurane inhalation (Forane, JW Pharmaceutical, Korea), after collecting their blood and euthanized by exsanguination from the posterior vena cava and aorta. Then necrosis was performed. A complete gross examination was performed on all terminated animals.

*(4) Organ Weights.* When we euthanized the animals, absolute organ weights were measured to calculate their relative organ weights (organ weight to fasted B.W. ratios before necropsy, %).

*(5) Hematological and Biochemical Parameters.* All the animals fasted all night before blood was taken. Blood samples were taken from the anesthetized aorta and transported to CBC bottles (EDTAK2, BD, USA) and vacutainer (9NC sodium citrate, BD, USA), respectively, for hematological and coagulation testing. Using centrifugation technique, the coagulation test separated the plasma (3,000 rpm, 4°C, 10 min). The following hematological parameters were measured by automated hematology analyzers (ADVIA 2120i, Siemens, USA) and coagulation analyzer (ACL 7000, Instrumentation Laboratory, USA): white blood cell count (WBC), differential count (neutrophils, lymphocytes, monocytes, basophils), red blood cell count (RBC), hemoglobin concentration (HGB), hematocrit (HCT), mean corpuscular volume (MCV), mean corpuscular hemoglobin (MCH), mean corpuscular hemoglobin concentration (MCHC), reticulocyte (Retic), platelets (PLT), prothrombin time (PT), and activated partial thromboplastin time (APTT). Except for blood samples for hematological and coagulation tests, the remaining samples were placed in an anticoagulant-free tube for serum separation. The tubes were kept at room temperature and the serum was separated by centrifugation (3,000 rpm, 4°C, 10 min). The following biochemical parameters were analyzed using an autoanalyzer (TBA-120FR, Toshiba, Japan): total protein (TP), albumin (ALB), A/G ratio, total bilirubin (T-BIL), alanine aminotransferase (ALT), aspartate aminotransferase (AST), alkaline phosphatase (ALP), creatinine (CREA), blood urea nitrogen (BUN), total cholesterol (T-CHO), triglycerides (TG), glucose (GLU), calcium (CA), inorganic phosphorus (IP), creatine kinase (CK), sodium (Na+), potassium (K+), and chloride (Cl-).

*(6) Histopathological Examination.* Organs and structures: liver, kidney, heart, brain, spleen, adrenal gland, lung and bronchus, pituitary gland, testis, epididymis, seminal vesicle, prostate gland uterus, ovary, vagina, tongue, trachea, esophagus, thyroid gland, parathyroid gland, thymus, stomach, small and large intestine, urinary bladder, salivary gland, pancreases, sternum, femur, eyeball, spinal cord, mesenteric lymph node, peripheral nerve, and skeletal muscle were extracted and examined. Tissues of each organ were fixed with 10% neutral buffered formalin, of which testes and epididymides were fixed with Bouin's fixative, and eyes with Harderian gland were fixed with Davidson's solution. Fixed liver, spleen, kidney, heart, and lungs of all the rats used in the highest dosage group and a control group were embedded in paraffin, sectioned, stained with hematoxylin and eosin (H&E stain), and examined microscopically.

### 2.7. Statistical Analysis

The data during the study period was analyzed by the SPSS program (ver. 19.0) and presented as mean ± SD (standard deviation) for body weight, food consumption, organ weight, hematology, and biochemistry. Leven's test was applied to the homogeneity of variance and one-way ANOVA was performed to evaluate the significant differences between groups. No further analysis was performed without significant differences, but if significant differences were confirmed, the post hoc test was performed according to variance homogeneity (Scheffe test). The significance level was considered at values of *p* < 0.05.

## 3. Results

### 3.1. Study of Acute Oral Toxicity

There were no deaths, clinical signs, and body weight changes in any dose groups (Table 2). Additionally, no gross lesions were found in any of the organs at necropsy. Based on these results of acute oral toxicity tests, approximate lethal dose (ALD) of HAD-B1 was considered to exceed 2000 mg/kg BW. for both sexes. Therefore, the dosage levels of 500, 1000, and 2000 mg/kg BW were selected for the DRF and subchronic toxicity studies.

### 3.2. Study of Dose Range Finding (DRF, 4-Week)

During the study period, treatment-related mortality, clinical signs, body weight change, and gross lesion were not found in any dose group in rats. In organ weight, significant differences were founded in the liver (2000 mg/kg, absolute weight) and prostate gland (500 mg/kg, absolute/relative weight) of males. In hematology and biochemistry, significant differences were observed in T-CHO (500 mg/kg) and MCV (2000 mg/kg) of females. However, the observed changes were minor levels within the historical data range and were not dose-dependent. Also, no test-substance-related histopathological examination was observed in the control and high-dose groups. Based on these results, the dosage levels of 500, 1000, and 2000 mg/kg BW were selected for the subchronic toxicity determined.

### 3.3. Study of Subchronic Toxicity (13-Week)

#### 3.3.1. Clinical Signs and Mortality

No clinical signs and mortality were observed in the treatment group (500, 1000, and 2000 mg/kg BW) for the 13-week treatment and 4-week recovery period. However, salivation was temporarily observed on days 89 to day 91 in one male and two female rats in the 2000 mg/kg BW group.

#### 3.3.2. Body Weight and Food Consumption

**Figure 3:**
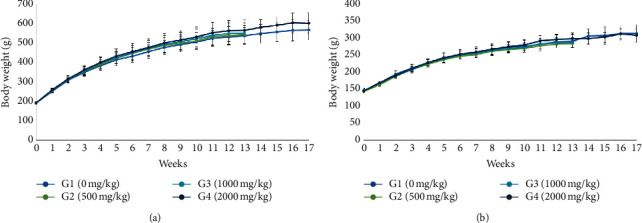
Body weight of male (a) and female (b) rats treated with HAD-B1 for 13-week treatment period and 4-week recovery period (mean ± SD). An increase tendency was showed in male (a). All groups showed increased body weight and there were no difference between each group (male).

No significant differences in mean body weight and food consumption were observed in the treatment group (500, 1000, and 2000 mg/kg BW) for the 13-week treatment period and 4-week recovery period. However, an increased tendency of body weight was observed during the treatment period and was continuously observed during the recovery period in 2000 mg/kg of males (Figures 2 and 3).

#### 3.3.3. Ophthalmological and Urine Analysis

No side effects related to the testing substance were observed in ophthalmological examination and urine test at any dose group.

#### 3.3.4. Gross Finding at Necropsy

**Figure 4:**
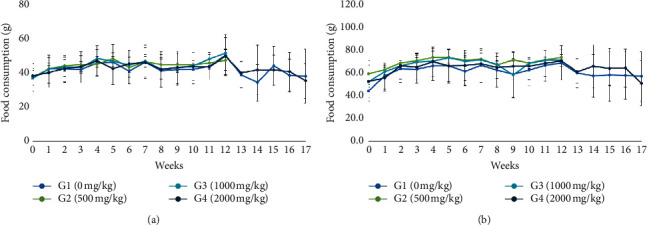
Food consumption of male (a) and female (b) rats treated with HAD-B1 for 13-week treatment period and 4-week recovery period (mean ± SD). All groups showed food and water consumption data, which indicated that there were no statistically significant differences among treated groups compared with the control group.

At the termination of treatment, no treatment-related gross finding was observed in the control and treatment group (500, 1000, and 2000 mg/kg BW) for 13-week study period including 4-week recovery period. However, reduced testis was observed in one animal at 2000 mg/kg of male and mass on the right mammary gland was observed in one animal in the control group of female (3.5 × 2.5 × 2.0) (Figure 4). The other gross findings were not observed.

#### 3.3.5. Organ Weights

In the treatment group, the absolute organ weight of the thymus was increased in 2000 mg/kg of males. In the recovery group, the relative organ weight of the spleen decreased in males, and the absolute/relative organ weight of the thymus was decreased in 2000 mg/kg of females. Other than that, no significant difference was observed (Tables 3 and 4 ).

#### 3.3.6. Hematological and Biochemical Parameters

There were no statistically significant differences in the hematological examination for the 13-week treatment period and 4-week recovery period at any dose group in males and females. In terms of biochemistry, A/G level was increased at 500 mg/kg in male as compared with the control group, but this increase was not observed at 1000 mg/kg, 2000 mg/kg in male and any dose group in female treated with test substance for 13-week treatment period (Table 5). In the recovery group, significant decreases were observed in T-BIL, ALP, AST, and ALT in males, and increases were observed in ALB, A/G in females (Table 6). However, this parameter showed no significant difference in the 13-week.

#### 3.3.7. Histopathological Examination

**Figure 5:**
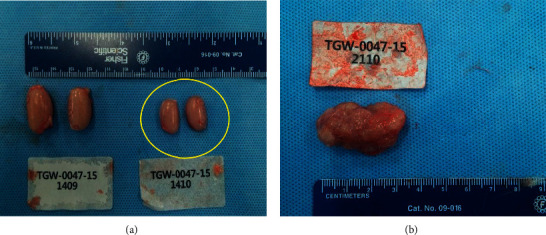
Gross finding at necropsy. (a) Reduced testis of rat treated with 2000 mg/kg in male (animal no. 1410). (b) Mass on the right mammary gland of untreated control rats (animal no. 2110). ○ indicates a reduced size of bilateral testis.

**Figure 6:**
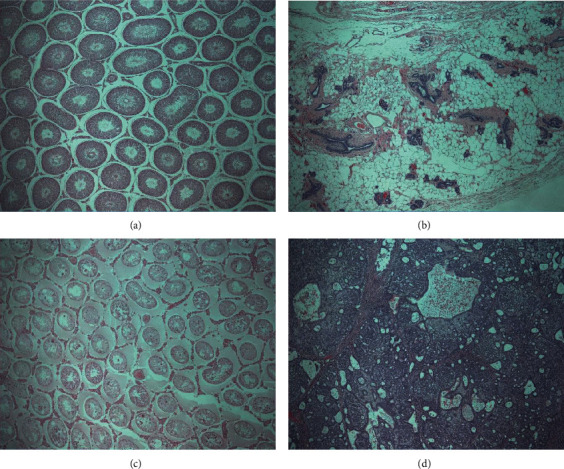
Micrographs of the testis and mammary glands sections (x50). (a, c) Normal histological section. (b) Reduced testis of rat treated with 2000 mg/kg in male (animal no. 1410). (d) Mass on the right mammary gland of untreated control rats (animal no. 2110). (a) Normal histological section of testis. (b) Tubular atrophy of testis. (c) Normal histological section of mammary gland. (d) Adenocarcinoma of mammary gland.

The histopathological changes were observed in high-dose group: inflammatory cell infiltration of the liver, tubular basophilia of kidneys, cortical vacuolation of the adrenal gland, inflammatory cell infiltration of the prostate gland at treatment group, and tubular basophilia/medullary cyst of a kidney, cortical vacuolation in the adrenal gland (Tables 7 and 8 ). Some lesions were found in the control group and/or were low in frequency. In addition, histopathological lesions were observed in organs (testis, mammary gland) where gross lesions were observed at necropsy: tubular atrophy in the testis of male, adenocarcinoma in a mass of mammary gland of female (Figures 5 and 6 ).

## 4. Discussion

Goals for any nonclinical safety study are identifying potential target organs for toxicity, evaluating potential reversibility toxicity, and determining the no observed adverse effect level (NOAEL) or no practical effect level [8]. NOAEL is the highest dose that produces no significant adverse effects in animals in a specific nonclinical toxicology study [9]. In this present research, acute toxicity, DRF, and subchronic toxicity studies for HAD-B1 were performed in SD rats (rodents) to investigate its potential toxicity in humans.

During the study, temporal hypersalivation was observed in 2000 mg/kg BW group in both male and female rats for acute toxicity and 4-week DRF studies. However, it was not a consistent manifestation and there was no sign of hypersalivation in any other animals. Thus, this observation resulted in a nonspecific response in those animals instead of a treatment-related effect.

Change in BW can be an adverse effect of drugs and chemicals [10]. As BW increased over time during both the treatment and recovering periods, this change was considered treatment-related. Thus, further study, e.g., a chronic toxicity study over 13 weeks, appears necessary to ascertain the toxic effects, if any, of HAD-B1 on BW.

Organ weight changes may occur when drugs or chemicals are used, and these changes could be meaningful indicators of test article-related changes, irrespective of corresponding microscopic findings, in repeated-dose rodent studies [11]. Although we observed a statistically significant increase in the absolute weight of the thymus in male rats in the 2000 mg/kg BW group during the subchronic toxicity study and statistically significant decreases during the 4-week recovery period in the relative organ weights of the thymus (female) and spleen (male) in rats in this group, other inter-group differences in organ weight either were minor or exhibited no dose-dependence. Furthermore, no treatment-related histological alterations were observed. Therefore, these changes were attributed to normal biological variations.

Blood parameters analysis can be used to determine the extent of foreign compounds' adverse effects [12]. In this research, clinical biochemistry analyses were conducted to evaluate possible alterations in blood chemistry and detect possible treatment-related changes compared with the control findings. A previous study identified an increased risk of anemia during a subchronic toxicity test, in contrast to our findings of no significant blood parameter changes [13]. Similarly, the changes in parameters during the study period, despite reaching significance, were minor, within the historical ranges, or not dose-dependent, illustrating that HAD-B1 had no significant toxic effects in SD rats.

Gross necropsy findings revealed reduced testis size in one male rat in the 2000 mg/kg BW group and a mass in the right mammary gland of a control female rat. Additionally, the histopathological findings revealed bilateral tubular atrophy of testis with oligospermia in the epididymis and adenocarcinoma (ductal subtype) in the mammary gland. Tubular atrophy is spontaneous [14], and oligospermia is considered a secondary effect of tubular atrophy. In general, classifying mammary adenocarcinomas by subtype (i.e., papillary, ductal, alveolar) in routine rodent carcinogenicity studies has not been demonstrated to be biologically meaningful. Besides, spontaneous mammary carcinomas typically occur in older rats, and they have been sporadically reported in control females as young as 10 weeks [9]. Therefore, these lesions were not considered treatment-related.

Based on this study's results, the NOAEL for HAD-B1 was considered >2000 mg/kg BW. In terms of dose translation from animals to humans, the human equivalent dose (HED; animal dose (mg/kg) × (animal Km factor/human Km factor)) calculated using the body surface area (mg/m^2^) for initial clinical trials in healthy adult volunteers [15, 16] was 324.32 mg/kg/day for adults (60 kg) and 480 mg/kg/day for children (20 kg). In the future, this HED may be evaluated in a clinical study.

## 5. Conclusions

In this study, the oral administration of HAD-B1 on the acute toxicity study and subchronic toxicity study in SD rats caused neither mortality nor any adverse effects noticed in clinical signs, body weight (gain), organ weight, and biochemical parameters. Also, the food consumption, urine analysis, and hematological parameters of HAD-B1 treated rats showed no significant changes than control, and there are no findings related to treatment given in necropsy finding and histological examination. Therefore, these results observed at the treatment doses (500, 1000, 2000 mg/kg BW) suggest that HAD-B1 is not likely to produce any toxicological effects and is safe for herbal medicinal use. However, since the experiment resulted in some weight gain, further study is considered to ascertain this mechanism.

## Figures and Tables

**Table 1:** Ingredients of HAD-B1.

Scientific name	Relative amount (g)
*Panax Notoginseng Radix*	25.2
*Cordyceps militaris*	19.2
*Panax ginseng* C.A. Mey.	19.2
*Boswellia carterii* Birdwood	14.4
Total amount	78.0

**Table 2:** Grouping of rats and weight in acute oral toxicity.

Rats						
Group	Dose (mg/kg BW)	Sex	*N*	Days after administration		
				0	7	14
G1	0	Male	5	163.5 ± 4.5	241.5 ± 5.0	305.9 ± 10.2
		Female	5	124.6 ± 4.4	166.3 ± 5.4	186.4 ± 06.3
G2	500	Male	5	161.5 ± 5.8	242.5 ± 6.3	311.0 ± 05.9
		Female	5	125.6 ± 7.0	171.5 ± 5.2	198.3 ± 08.2
G3	1000	Male	5	158.4 ± 6.0	232.2 ± 8.4	303.6 ± 15.4
		Female	5	127.9 ± 4.8	174.4 ± 3.8	197.0 ± 05.7
G4	2000	Male	5	156.7 ± 3.6	233.4 ± 8.4	293.8 ± 11.1
		Female	5	125.0 ± 1.7	171.3 ± 7.0	196.2 ± 09.4
		Female	3	127.9 ± 4.8	174.4 ± 3.8	197.0 ± 05.7

*N*: number of animals.

**Table 3:** Absolute organ weights of male rats treated with HAD-B1 for a 13-week period.

Group		Liver		Kidney		Heart	Brain	Spleen	Testis		Epididymis		PG	SG	Lung	Thymus	Adrenal gland		Pituitary gland
				L	R				L	R	L	R					L	R	
Male	G1 (0)	Mean	13.20	1.69	1.66	1.69	2.20	0.82	1.76	1.75	0.89	0.87	0.97	0.84	1.81	0.40	0.039	0.036	0.019
		SD	1.42	0.08	0.10	0.12	0.05	0.07	0.16	0.15	0.09	0.08	0.33	0.07	0.12	0.07	0.004	0.006	0.006
	G2 (500)	Mean	13.39	1.71	1.73	1.72	2.23	0.89	1.75	1.75	0.87	0.85	0.95	0.83	1.90	0.44	0.038	0.033	0.018
		SD	1.86	0.19	0.21	0.19	0.10	0.18	0.10	0.12	0.12	0.12	0.25	0.08	0.19	0.10	0.011	0.009	0.004
	G3 (1000)	Mean	13.06	1.71	1.73	1.62	2.29	0.86	1.77	1.79	0.89	0.86	0.80	0.81	1.91	0.44	0.036	0.033	0.019
		SD	1.93	0.17	0.15	0.13	0.13	0.16	0.14	0.10	0.11	0.06	0.19	0.15	0.19	0.09∗	0.006	0.006	0.005
	G4 (2000)	Mean	14.56	1.80	1.81	1.82	2.27	0.90	1.74	1.76	0.88	0.91	0.94	0.92	2.00	0.51	0.037	0.039	0.017
		SD	1.57	0.16	0.12	0.16	0.19	0.08	0.29	0.33	0.12	0.13	0.27	0.12	0.26	0.08	0.01	0.01	0.004

Group		Liver		Kidney		Heart	Brain	Spleen	S.G	Lung	Thymus	Uterus			Adrenal gland		Ovary		Pituitary gland
				L	R										L	R	L	R	

Female	G1 (0)	Mean	7.28	1.00	1.00	1.03	2.04	0.52	0.55	1.37	0.32	0.86			0.041	0.040	0.057	0.057	0.023
		SD	0.63	0.10	0.07	0.13	0.10	0.04	0.06	0.17	0.04	0.28			0.004	0.004	0.019	0.017	0.006
	G2 (500)	Mean	6.99	0.96	0.98	0.97	1.98	0.50	0.52	1.33	0.34	0.80			0.043	0.040	0.054	0.099	0.024
		SD	0.36	0.09	0.07	0.07	0.09	0.05	0.06	0.14	0.05	0.16			0.009	0.007	0.013	0.159	0.007
	G3 (1000)	Mean	7.22	0.96	0.99	0.98	2.04	0.54	0.52	1.37	0.32	0.73			0.042	0.041	0.051	0.051	0.025
		SD	0.86	0.09	0.09	0.09	0.08	0.06	0.03	0.11	0.06	0.14			0.009	0.008	0.014	0.019	0.006
	G4 (2000)	Mean	7.64	0.98	0.98	1.05	2.05	0.58	0.58	1.43	0.34	0.72			0.041	0.044	0.049	0.060	0.052
		SD	0.75	0.08	0.08	0.09	0.10	0.05	0.11	0.11	0.06	0.14			0.005	0.006	0.010	0.012	0.080

Number of animals: 10; SD: standard deviation; PG: prostate gland; SG: salivary gland; unit: g. *∗*Significant difference as compared with control, *p* < 0.05.

**Table 4:** Relative organ weights (organ weight/body weight, %) of rats for 4-week recovery period.

Group		Liver		Kidney		Heart	Brain	Spleen	Testis		Epididymis		PG	SG	Lung	Thymus	Adrenal gland		Pituitary gland
				L	R				L	R	L	R					L	R	
Male	G1 (0)	Mean	2.36	0.35	0.34	0.32	0.43	0.18	0.34	0.34	0.18	0.18	0.15	0.16	0.38	0.09	0.006	0.005	0.003
		SD	0.22	0.02	0.03	0.01	0.03	0.02*∗*	0.02	0.02	0.02	0.01	0.02	0.02	0.03	0.01	0.001	0.001	0.001
	G4 (2000)	Mean	2.51	0.33	0.36	0.30	0.40	0.14	0.31	0.31	0.16	0.16	0.14	0.15	0.36	0.08	0.007	0.006	0.003
		SD	0.18	0.004	0.004	0.01	0.04	0.01	0.04	0.05	0.02	0.02	0.04	0.01	0.05	0.01	0.002	0.002	0.001

Group		Liver		Kidney		Heart	Brain	Spleen	S.G	Lung	Thymus	Uterus			Adrenal gland		Ovary		Pituitary gland
				L	R										L	R	L	R	

Female	G1 (0)	Mean	2.50	0.34	0.36	0.33	0.69	0.17	0.18	0.48	0.11	0.22			0.014	0.013	0.016	0.018	0.008
		SD	0.17	0.02	0.03	0.03	0.08	0.02	0.02	0.03	0.01∗	0.04			0.002	0.002	0.002	0.002	0.002
	G4 (2000)	Mean	2.59	0.32	0.34	0.36	0.69	0.18	0.18	0.48	0.08	0.29			0.013	0.013	0.015	0.016	0.007
		SD	0.09	0.04	0.04	0.03	0.05	0.02	0.02	0.02	0.01	0.08			0.002	0.001	0.002	0.003	0.002

Number of animals: 5; SD: standard deviation; PG: prostate gland; SG: salivary gland; unit: %. *∗*Significant difference as compared with control, *p* < 0.05. Number of animals: 5; SD: standard deviation; SC: salivary gland; unit: %. *∗*Significant difference as compared with control, *p* < 0.05.

**Table 5:** Biochemical values of rats treated with HAD-B1 for 13-week period.

Group (mg/kg)			TP (g/dL)	ALB (g/dL)	A/*G* ratio	T-BIL (mg/dL)	ALP (U/L)	AST (U/L)	ALT (U/L)	CREA (mg/dL)	BUN (mg/dL)	T-CHO (mg/dL)	TG (mg/dL)	GLU (mg/dL)	CA (mg/dL)	IP (mg/dL)	CK (U/L)	Na (mmol/L)	K (mmol/L)	Cl (mmol/L)
Male	G1 (0)	Mean	5.9	3.8	1.8	0.03	279.9	97.7	36.0	0.5	17.8	64.3	26.2	170.4	9.1	5.7	273.3	146.0	4.37	106.9
		SD	0.3	0.1	0.1	0.01	42.3	19.2	3.6	0.0	3.4	19.8	7.8	22.1	0.3	0.4	215.8	0.7	0.39	1.2
	G2 (500)	Mean	5.8	3.8	1.9	0.03∗	274.3	93.3	33.9	0.5	16.2	56.4	37.5	169.8	9.1	5.7	304.9	146.2	4.53	106.9
		SD	0.2	0.1	0.1	0.02	30.8	15.3	4.5	0.1	2.5	11.3	14.1	23.1	0.3	0.6	176.4	1.2	0.38	1.5
	G3 (1000)	Mean	6.8	4.4	1.9	0.1	147.5	125.4	44.7	0.6	20.8	78.4	18.3	143.8	9.7	4.5	449.9	145.0	4.1	107.4
		SD	0.6	0.4	0.1	0.0	50.6	38.8	22.3	0.1	4.4	24.9	8.2	12.6	0.6	1.2	326.5	1.2	0.4	2.9
	G4 (2000)	Mean	6.7	4.4	2.0	0.1	133.3	113.9	38.5	0.5	19.5	75.7	21.3	143.7	9.7	4.8	377.3	145.2	4.1	107.2
		SD	0.5	0.4	0.1	0.0	21.2	76.6	23.8	0.0	1.7	23.0	9.1	13.1	0.4	0.9	257.1	0.8	0.5	1.9

Female	G1 (0)	Mean	6.5	4.3	2.0	0.1	137.9	108.3	39.3	0.5	19.7	75.9	15.6	148.5	9.6	4.6	261.7	145.2	4.1	107.4
		SD	0.5	0.4	0.2	0.0	38.5	36.6	16.4	0.0	2.8	18.8	5.7	17.5	0.4	1.0	240.2	1.6	0.3	1.8
	G2 (500)	Mean	6.4	4.2	1.9	0.1	185.1	102.5	33.9	0.5	18.7	67.3	17.9	145.6	9.4	4.7	283.9	145.4	4.1	107.1
		SD	0.3	0.2	0.1	0.0	45.3	22.5	7.9	0.0	2.7	11.6	6.3	18.4	0.2	0.9	152.3	0.6	0.2	1.2
	G3 (1000)	Mean	6.8	4.4	1.9	0.1	147.5	125.4	44.7	0.6	20.8	78.4	18.3	143.8	9.7	4.5	449.9	145.0	4.1	107.4
		SD	0.6	0.4	0.1	0.0	50.6	38.8	22.3	0.1	4.4	24.9	8.2	12.6	0.6	1.2	326.5	1.2	0.4	2.9
	G4 (2000)	Mean	6.7	4.4	2.0	0.1	133.3	113.9	38.5	0.5	19.5	75.7	21.3	143.7	9.7	4.8	377.3	145.2	4.1	107.2
		SD	0.5	0.4	0.1	0.0	21.2	76.6	23.8	0.0	1.7	23.0	9.1	13.1	0.4	0.9	257.1	0.8	0.5	1.9

Number of animals: 10; SD: standard deviation. *∗*Significant difference as compared with control, *p* < 0.05.

**Table 6:** Biochemical values of rats for 4-week recovery.

Group (mg/kg)			TP (g/dL)	ALB (g/dL)	A/G ratio	T-BIL (mg/dL)	ALP (U/L)	AST (U/L)	ALT (U/L)	CREA (mg/dL)	BUN (mg/dL)	T-CHO (mg/dL)	TG (mg/dL)	GLU (mg/dL)	CA (mg/dL)	IP (mg/dL)	CK (U/L)	Na (mmol/L)	K (mmol/L)	Cl (mmol/L)
Male	G1 (0)	Mean	5.8	3.7	1.8	0.04	248.2	117.0	46.0	0.5	13.1	58.0	25.8	158.6	8.7	4.8	275.2	146.5	4.4	107.1
		SD	0.2	0.1	0.2	0.01*∗*	11.8*∗*	25.6	6.2	0.1	0.7	6.4	8.1	13.2	0.2	0.2	163.8	0.4	0.3	1.0
	G4 (2000)	Mean	5.8	3.7	1.8	0.01	208.2	87.4*∗*	34.0*∗*	0.4	12.0	56.8	28.8	175.6	8.8	5.0	190.6	145.6	4.3	106.2
		SD	0.3	0.2	0.1	0.01	34.3	12.7	2.8	0.1	1.1	13.2	12.4	12.2	0.2	0.4	120.8	1.1	0.3	1.6

Female	G1 (0)	Mean	6.3	4.1	1.9	0.03	119.0	87.6	37.6	0.5	17.2	62.0	18.2	178.2	9.2	4.8	186.4	146.7	4.5	107.5
		SD	0.2	0.1	0.2*∗*	0.01	14.7	4.8	7.0	0.0	1.6	6.4	5.8	17.1	0.1	0.4	46.0	1.6	0.3	0.6
	G4 (2000)	Mean	6.4	4.3*∗*	2.1	0.06	124.6	87.2	42.8	0.5	14.8	68.4	20.8	173.0	9.0	4.5	180.2	146.7	4.1	107.6
		SD	0.2	0.1	0.1	0.02	12.3	16.7	25.4	0.1	2.3	10.4	7.6	18.5	0.3	0.5	66.0	0.7	0.3	1.5

Number of animals: 5; SD: standard deviation. *∗*Significant difference as compared with control, *p* < 0.05.

**Table 7:** Histopathological findings of rats treated with HAD-B1 for 13-week period.

Organ	*N*	Histological findings	Male		Female	
			G1	G4	G1	G4
Liver	10	Inflammatory cell foci
		Minimal, multifocal	2	2	0	1
Kidney	10	Tubular basophilia
		Minimal, multifocal	3	2	0	0
		Minimal, focal	0	1	0	0
		Slight, multifocal	0	1	0	0
		Tubular casts
		Present	1	0	0	0
		Cortical scar
		Present	1	0	2	0
		Medullary mineralization
		Minimal, multifocal	1	0	2	0
Adrenal gland	10	Cortical vacuolation
		Minimal, diffuse	2	2	0	0
Lung	10	Inflammatory cell infiltration, perivascular
		Minimal, multifocal	2	0	0	0
Prostate gland		Inflammatory cell infiltration, interstitial			—	—
		Minimal, multifocal	2	1		
		Slight, multifocal	0	1		
		Concretion				
		Minimal, multifocal	1	0		
Testis	10	Bilateral tubular atrophy			—	—
		Severe, diffuse	0	1∗		
Epididymis	10	Oligospermia, bilateral			—	—
		Moderate, diffuse	0	1		
Thyroid gland	10	Ultimobranchial cysts				
		Present	1	0	0	0
Mammary gland	10	Adenocarcinoma	—	—		
		Present			1∗∗	0

—: not applicable; *∗*: reduced testis at necropsy; *∗∗*: mass on the right mammary gland at necropsy.

**Table 8:** Histopathological findings of rats for 4-week recovery.

Organ	*N*	Histological findings	Male		Female	
			G1	G4	G1	G4
Liver	5	Inflammatory cell foci				
		Minimal, multifocal	0	0	1	0
Kidney	5	Tubular basophilia				
		Minimal, multifocal	1	1	0	0
		Medullary cyst				
		Present	0	1	0	0
Adrenal gland	5	Cortical vacuolation				
		Slight, diffuse	0	1	0	0
Heart	5	Inflammatory cell infiltration				
		Minimal, multifocal	1	0	0	0
Lung	5	Alveolar macrophage infiltration				
		Minimal, multifocal	1	0	0	0

## Data Availability

The data used to support the findings of this study are included within the article.
